# A Novel Nomogram for the Early Identification of Cardioembolic Stroke Using Clinical and Dual-Energy CT Features

**DOI:** 10.31083/RCM37447

**Published:** 2025-08-25

**Authors:** Yao Dai, Ying Zhao, Ziyang Song, Yuzhu Ma, Jiajia Yang, Yu Zhang

**Affiliations:** ^1^Department of Radiology, The Fourth Affiliated Hospital of Soochow University (Suzhou Dushu Lake Hospital), 215124 Suzhou, Jiangsu, China; ^2^Department of Radiology, The First Affiliated Hospital of Soochow University, 215006 Suzhou, Jiangsu, China

**Keywords:** acute ischemic stroke, cardioembolic stroke, thrombosis, dual-energy CT, nomogram

## Abstract

**Background:**

Identifying the etiology of acute ischemic stroke (AIS) is critical for secondary prevention and treatment choice in stroke patients. This study aimed to investigate the dual-energy computed tomography (DECT) quantitative thrombus parameters associated with cardioembolic (CE) stroke and develop a nomogram that combines DECT and clinical data to identify CE stroke.

**Methods:**

We retrospectively reviewed all consecutive patients from January 2020 to July 2022 with anterior circulation stroke and proximal intracranial occlusions. Patients were divided into CE stroke and non-cardioembolic (NCE) stroke groups according to the Trial of Org 10172 in Acute Stroke Treatment (TOAST) criteria. Univariable and multivariable logistic analyses were conducted, and a nomogram was developed by combining clinical and DECT variables. This nomogram was subsequently validated using an independent internal cohort of patients.

**Results:**

A total of 96 patients were analyzed, of which 43 (45%) were diagnosed with CE stroke. The multivariable analysis identified the following factors as being independently associated with CE stroke: normalized iodine concentration (NIC) (per 10^-2^ unit increase) (odds ratio (OR) = 1.598, 95% CI: 1.277–1.998; *p* < 0.001), gender (OR = 0.113, 95% CI: 0.028–0.446; *p* = 0.002), hypertension (OR = 0.204, 95% CI: 0.054–0.770; *p* = 0.019), and baseline National Institutes of Health Stroke Scale (NIHSS) (OR = 1.168, 95% CI: 1.053–1.296; *p* = 0.003). The matching nomogram displayed an area under the curve (AUC) of 0.929 in the study sample (n = 96) and 0.899 in the validation cohort (n = 29).

**Conclusions:**

A nomogram that combines clinical and DECT variables can display good diagnostic performance for CE stroke.

## 1. Introduction

Acute ischemic stroke (AIS) is one of the major diseases causing disability and death. Ischemic stroke, especially intracranial large blood vessel occlusion, imposes a large financial burden on society and families due to its poor prognosis [1]. Identifying the etiology of AIS is therefore of great significance for the selection of secondary prevention measures and treatment choices. For secondary prevention, anticoagulant drugs such as warfarin are preferred for cardioembolic (CE) [2] stroke. Compared to non-cardioembolic (NCE) thrombi, CE thrombi are increasingly considered to be more difficult to remove, with catheter aspiration often used as a supplemental method to stent retrieval during mechanical thrombectomy [3, 4, 5, 6]. This may be due to a stiffer thrombus and greater friction coefficient [7]. It is therefore important to identify the source of thrombi early, allowing for timely treatment with mechanical thrombectomy. Atrial fibrillation (AF) is the main cause of CE stroke, but some AF is not discovered until the left atrial appendage thrombi are shed off. No documented prior history of AF increases the difficulty in identifying CE stroke at admission due to the limitation of emergency electrocardiograms (ECGs) on detecting paroxysmal AF. CE stroke can also be triggered by other cardiogenic causes, such as patent foramen ovalis. Therefore, other indicators besides AF are needed to identify CE stroke.

Some readily available clinical indicators can help to diagnose the etiology of stroke. CE stroke is more common in women with high baseline National Institutes of Health Stroke Scale (NIHSS) [8, 9, 10, 11]. Patients with hypertension often progress to NCE, mainly large artery atherosclerosis (LAA) stroke. Compared to NCE thrombi, CE thrombi usually have higher thrombus permeability, which indicates the ability of contrast agents to permeate the irregular space in the thrombus [12, 13]. Conventional permeability is evaluated as the increased thrombus attenuation between noncontrast computed tomography (NCCT) and CT angiography (CTA) images [14, 15]. This method requires precise co-registration, which is time-consuming and difficult to achieve in clinical practice, as well as being prone to inevitable measurement error [16]. Dual-energy CT (DECT) generates iodine overlay maps based on material decomposition technology. It can directly obtain the penetration amount of the contrast agent by displaying the iodine concentration (IC) in the thrombus [17, 18, 19]. The IC of the thrombus was normalized to the IC of the contralateral normal vessel to derive a normalized IC (NIC) value, thereby eliminating the potential effect of individual circulation differences on the IC of the lesion. The NIC of the thrombus is a new permeability parameter that is assessed using iodine overlay maps alone without the co-registration process. Therefore, we hypothesized that NIC may have higher clinical value for identifying CE stroke, with a simpler operation process and less measurement error. In addition, previous studies have suggested that there were differences in the thrombus composition between CE and NCE stroke [13, 20, 21, 22]. CE thrombi consist of fewer red blood cells (RBCs) and more fibrin/platelet (FP) conglomerations, while the opposite is seen for NCE thrombi. DECT allows the operator to analyze how the material-specific attenuation varies with X-ray photon energy [23]. We suspected that DECT could distinguish between thrombi with similar X-ray attenuation but different histopathologic compositions by atomic numbers and spectral attenuation curves, as represented by the effective atomic number (Zeff) and slope (λ_Hounsfield unit [HU]_), respectively. To our knowledge, there have been no reports on the performance of DECT thrombus variables in combination with clinical data for the identification of CE stroke.

The aims of this study were therefore to identify quantitative DECT thrombus parameters that are associated with CE stroke, and to subsequently combine these variables with clinical data in a nomogram to identify CE stroke.

## 2. Methods

### 2.1 Subjects

**Fig. 1.**
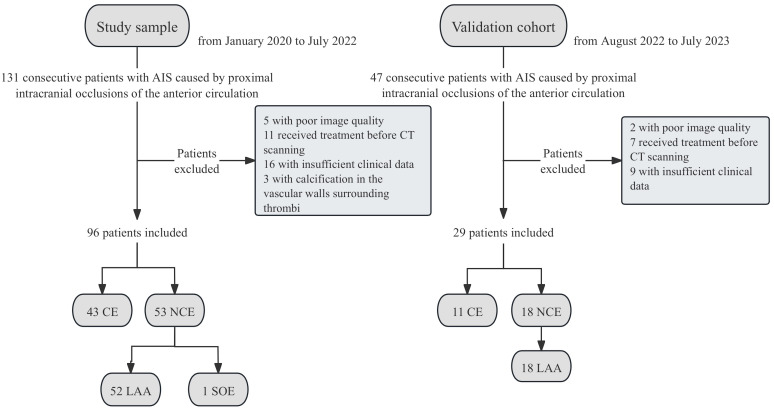
**Flowchart for enrollment of the study sample and validation cohort**. AIS, acute ischemic stroke; CE, cardioembolic; NCE, non-cardioembolic; LAA, atherosclerotic; SOE, other determined etiology; CT, computed tomography.

The study was approved by the hospital’s Ethics Committee and the requirement for individual consent was waived due to the retrospective design of the study (Approval number: 2022 the 59th). We retrospectively reviewed consecutive patients admitted to our hospital between January 2020 and July 2022 for acute anterior circulation stroke and proximal intracranial occlusions, and who received multimodal DECT scans at admission (n = 131 patients). The exclusion criteria were: (a) poor image quality (images with motion or metal artifacts that affected the measurements); (b) patients received treatment before CT scanning; (c) clinical data were insufficient to determine the sources of thrombi (patients without ECG-monitoring for at least 24 hours or echocardiogram); and (d) the CT showed calcification in the vascular walls surrounding thrombi which could interfere with thrombus assessment. Thirty-five patients were excluded based on these criteria, leaving 96 patients who were finally enrolled in the study. Fig. 1 presents a flowchart of the study enrollment.

### 2.2 Imaging Protocols

NCCT and dual-energy CTA (DECTA) were conducted upon admission using a 256-slice CT scanner (Revolution CT, GE Healthcare, Chicago, IL, USA). NCCT (conventional helical mode): tube voltage 120 kVp, tube current 320 mA. DECTA (Gemstone Spectral Imaging [GSI] mode): rapid tube voltage switching between 80 and 140 kVp within the duration of a single rotation, triggered when the CT numbers at the ascending aorta rose to 120 HU by utilizing an automated bolus-tracking technology, contrast agent (Iopamidol, 370 mg/mL, Bayer, Leverkusen, Germany) at 1 mL/kg of body weight injected at 5 mL/s. DECTA images were imported into the machine-equipped workstation (AW VolumeShare 7) for analysis. Iodine overlay maps, water overlay maps, and Zeff maps were generated with a 0.63 mm slice thickness. Spectral attenuation curves were also generated.

### 2.3 Imaging Analyses

**Fig. 2.**
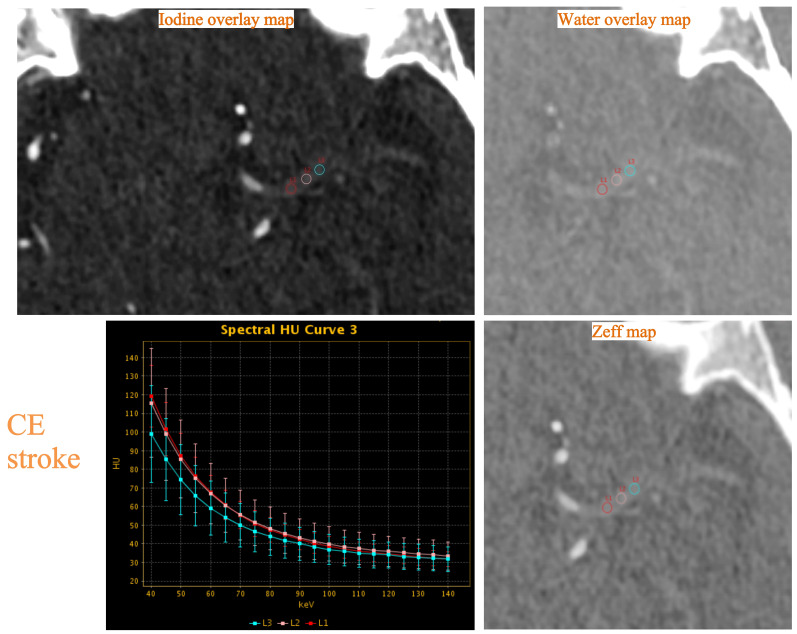
**Example of thrombus dual-energy CT (DECT) variables assessment in a 77-year-old woman with occlusion of the left middle cerebral artery and cardioembolic (CE) stroke**. The three ROIs on the thrombus were labeled as L1, L2, and L3. The normalized iodine concentration (NIC), water concentration (WC), effective atomic number (Zeff) and slope (λ_Hounsfield unit [HU]_) were measured on the iodine overlay map, water overlay map, Zeff map and spectral HU curve, respectively.

**Fig. 3.**
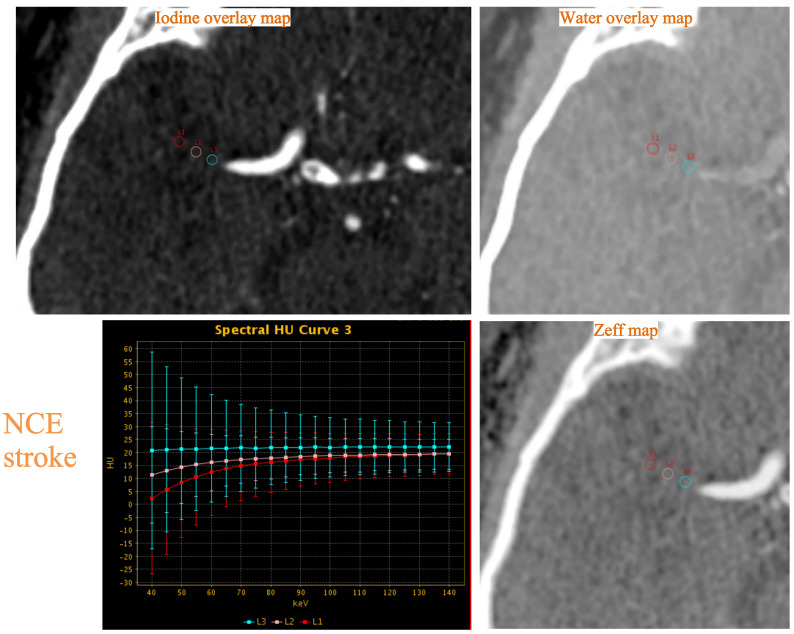
**Example of thrombus dual-energy CT (DECT) variables assessment in a 56-year-old man with occlusion of the right middle cerebral artery and non-cardioembolic (NCE) stroke**. The normalized iodine concentration (NIC), water concentration (WC), effective atomic number (Zeff) and slope (λ_HU_) were measured on the iodine overlay map, water overlay map, Zeff map and spectral HU curve, respectively.

All DECTA images were assessed on the AW VolumeShare 7 workstation by two experienced and trained neuroradiologists who were blinded to the clinical data. Disagreements with regard to measurements were settled by consensus between the two readers. The NIC, water concentration (WC) and Zeff were measured on iodine overlay maps, water overlay maps, and Zeff maps, respectively. Zeff maps disaplayed the effective atomic number of the substance. The slope of the spectral attenuation curve (λ_HU_) was calculated as the ratio of the CT numbers difference at 40 and 100 keV to the energy difference of 60. This was calculated as follows: λ_HU_ = (CT_40keV_–CT_100keV_)/(100–40). Three regions of interest (ROIs) with a radius of 1 mm were positioned in the proximal, middle, and distal segments of the thrombus. The mean iodine concentration of three ROIs was used to represent the thrombus iodine concentration (IC_thrombus_). A small fraction of the thrombi were short, in which case one or two ROIs were positioned based on their length. The IC of the contralateral vessel (IC_contralateral_) was evaluated as a reference. The NIC, which is the ratio of the IC_thrombus_ to the IC_contralateral_ (NIC = IC_thrombus_/IC_contralateral_), was then calculated [19]. In addition to arterial-phase DECTA, delayed venous-phase CTA was also performed. Distal vessels beyond the thrombus may become opacified over time due to collateral circulation, thrombus permeability, etc. Therefore, delayed venous-phase CTA was utilized to identify the distal extent of the thrombus. If there was still doubt, CT hyperdense artery sign on NCCT images was also used to evaluate the thrombus extension. The ROIs were placed in the same positions as the NIC measurements on water overlay maps, Zeff maps, monochromatic 40-keV images and monochromatic 100-keV images. The WC, Zeff and λ_HU_ were calculated as the average of three ROIs. Examples of the quantitative parameters measurements of thrombi in two patients are described in Figs. 2,3.

### 2.4 Assessment of Stroke Etiology

Two experienced and trained observers assessed the stroke etiology according to the Trial of Org 10172 in Acute Stroke Treatment (TOAST) classification based on clinical data [24]. Patients were assigned to two groups according to this classification: CE stroke (TOAST 2) and NCE stroke (TOAST 1 and 4).

### 2.5 Statistical Analysis

Statistical analysis was performed using SPSS version 20.0 (IBM Corp, Armonk, NY, USA) and R version 4.1.3 (R Foundation for Statistical Computing, Vienna, Austria). Continuous variables were compared using the Student’s *t-*test or Mann Whitney *U* test, and categorical variables were compared using the χ^2^ test. Baseline characteristics were compared between patients with CE and NCE stroke. To evaluate the probability of CE stroke, variables with *p* < 0.10 in the univariable analysis were included in a multivariable logistic model with backward stepwise selection. The odds ratio (OR) and the corresponding 95% CI were calculated. A predictive nomogram for CE stroke was built according to the model. To assess the performance of the nomogram, both the discrimination (the ability to distinguish between CE and NCE stroke) and calibration (agreement between predicted and observed probabilities) were evaluated. The area under the curve (AUC) is the quantitative indicator of the receiver operator characteristic (ROC) curve and was used to assess the efficiency of the model. AUCs were compared by the Delong test, which determines the statistical significance of differences between AUC values. The CT numbers of thrombi were measured in HU. These ranged between 11.50 HU and 160.42 HU at 40 keV, and between 19.47 HU and 67.58 HU at 100 keV in the study sample.

The independent validation cohort consisted of 29 patients admitted consecutively to the same hospital between August 2022 and July 2023. Patients from this cohort met the same inclusion and exclusion criteria as patients from the study cohort.

All statistical tests were performed using a two-tailed approach, with the significance threshold defined as *p* < 0.05.

## 3. Results

### 3.1 Characteristics of the Study Cohort

Of 131 patients who were initially reviewed, 96 patients fulfilled the inclusion criteria and were subsequently enrolled in the study sample. The median age was 71 years (interquartile range [IQR], 58 to 76 years) and 61% (n = 59) of patients were men. Of the 96 patients, 43 (45%) had CE stroke, 52 (54%) had LAA stroke and 1 (1%) had stroke caused by other determined etiology (SOE). Table 1 presents the clinical and DECT characteristics of the CE and NCE stroke groups. Compared to patients with NCE stroke, patients with CE stroke had significantly greater NIC (0.104 ± 0.043 vs 0.051 ± 0.033, *p* < 0.001), Zeff (8.01 ± 0.17 vs 7.90 ± 0.21, *p* = 0.009), λ_HU_ (0.79 ± 0.32 vs 0.59 ± 0.37, *p* = 0.007), and baseline NIHSS (14 [IQR, 11 to 19] vs 8 [IQR, 4 to 13], *p* < 0.001). The CE stroke group also had a higher proportion of women (56% vs 25%, *p* = 0.002) and a more frequent history of AF (84% vs 6%, *p* < 0.001). In contrast, patients with NCE stroke had more frequent diabetes mellitus (32% vs 12%, *p* = 0.018), hypertension (72% vs 49%, *p* = 0.022), and smoking history (36% vs 9%, *p* = 0.002) compared to those with CE stroke (Table 1).

**Table 1.** **Characteristics of the study sample and validation cohort**.

Characteristic		Study sample			Comparison with validation cohort		
		CE group	NCE group	*p* value	Study sample	Validation cohort	*p* value
No. of patients, n (%)		43 (45)	53 (55)	-	96	29	-
Gender				0.002			0.693
	No. of women, n (%)	24 (56)	13 (25)		37 (39)	10 (34)	
	No. of men, n (%)	19 (44)	40 (75)		59 (61)	19 (66)	
Age (yr), median (IQR)		72 (62–76)	70 (57–76)	0.295	71 (58–76)	73 (60–80)	0.336
Baseline NIHSS, median (IQR)		14 (11–19)	8 (4–13)	<0.001	11 (5–17)	8 (4–13)	0.109
Time from symptom onset to CT (min), median (IQR)		180 (120–300)	180 (120–300)	0.759	180 (120–300)	223 (115–435)	0.381
Diabetes mellitus, n (%)		5 (12)	17 (32)	0.018	22 (23)	8 (28)	0.606
Hypertension, n (%)		21 (49)	38 (72)	0.022	59 (61)	21 (72)	0.281
Dyslipidemia, n (%)		23 (53)	33 (62)	0.386	56 (58)	17 (59)	0.978
Smoking history, n (%)		4 (9)	19 (36)	0.002	23 (24)	6 (21)	0.715
Atrial fibrillation, n (%)		36 (84)	3 (6)	<0.001	39 (41)	11 (38)	0.795
NIC, mean ± SD		0.104 ± 0.043	0.051 ± 0.033	<0.001	0.075 ± 0.046	0.068 ± 0.042	0.471
WC (mg/cm^3^), mean ± SD		1033.25 ± 6.74	1030.92 ± 8.77	0.156	1031.96 ± 7.97	1024.24 ± 7.19	<0.001
Zeff, mean ± SD		8.01 ± 0.17	7.90 ± 0.21	0.009	7.95 ± 0.20	7.94 ± 0.19	0.913
λ_HU_, mean ± SD		0.79 ± 0.32	0.59 ± 0.37	0.007	0.68 ± 0.36	0.67 ± 0.35	0.935

Abbreviations: CE, cardioembolic; NCE, non-cardioembolic; NIHSS, National Institutes of Health Stroke Scale; NIC, normalized iodine concentration; WC, water concentration; Zeff, effective atomic number; λ_HU_, slope of the spectral attenuation curve; IQR, interquartile range.

### 3.2 Multivariable Analysis

**Fig. 4.**
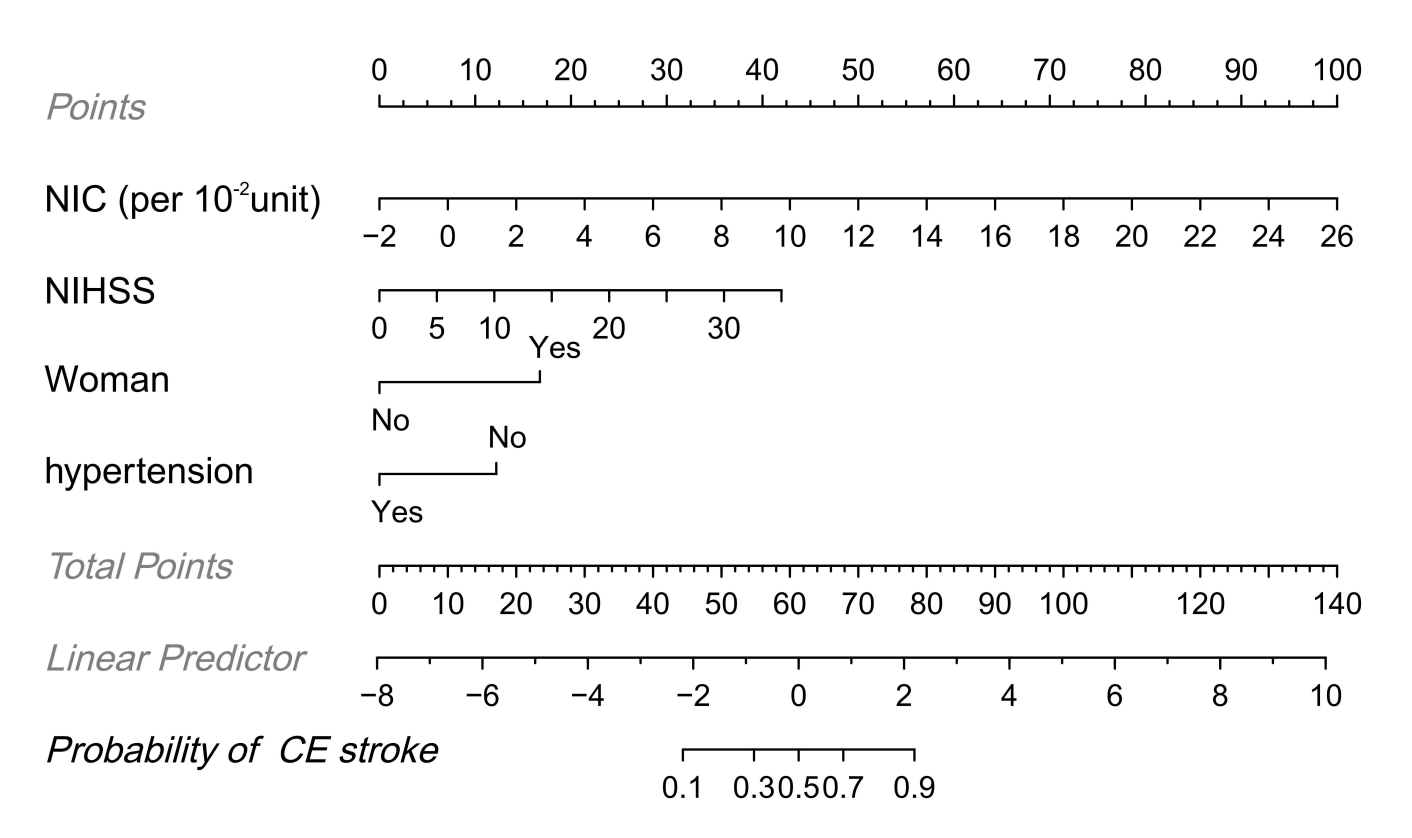
**Nomogram for the identification of cardioembolic (CE) stroke identification based on combined clinical and dual-energy CT (DECT) features**. Each factor was assigned a numerical value ranging from 0 to 100, with the total score being the sum of scores for all factors. The total score was mapped to the probability axis at the bottom of the model, providing a visual representation of the estimated probability of CE stroke.

Table 2 presents the findings of univariable and multivariable analysis of the baseline clinical and DECT variables. The multivariable analysis identified four variables that were significantly associated with CE stroke: NIC (per 10^-2^ unit increase) (OR = 1.598, 95% CI: 1.277–1.998, *p* < 0.001), gender (OR = 0.113, 95% CI: 0.028–0.446, *p* = 0.002), hypertension (OR = 0.204, 95% CI: 0.054–0.770, *p* = 0.019), and baseline NIHSS (OR = 1.168, 95% CI: 1.053–1.296, *p* = 0.003). These four variables were used to construct a nomogram for the identification of CE stroke (Fig. 4).

**Table 2.** **Univariable and multivariable analysis showing associations between baseline clinical variables, DECT variables, and CE stroke**.

	Univariable model		Multivariable model	
	Odds ratio (95% CI)	*p* value	Odds ratio (95% CI)	*p* value
Gender*	0.257 (0.108–0.613)	0.002	0.113 (0.028–0.446)	0.002
Baseline NIHSS	1.165 (1.080–1.257)	<0.001	1.168 (1.053–1.296)	0.003
Diabetes mellitus	0.279 (0.093–0.834)	0.022	…	0.364
Hypertension	0.377 (0.162–0.878)	0.024	0.204 (0.054–0.770)	0.019
Smoking history	0.184 (0.057–0.593)	0.005	…	0.540
NIC, per 10^-2^ unit increase	1.490 (1.267–1.752)	<0.001	1.598 (1.277–1.998)	<0.001
Zeff	17.751 (1.887–167.009)	0.012	…	0.317
λ _HU_	5.136 (1.487–17.743)	0.010	…	0.339

Abbreviations: NIHSS, National Institutes of Health Stroke Scale; NIC, normalized iodine concentration; Zeff, effective atomic number; λ_HU_, slope of the spectral attenuation curve. *Female is used as a reference. Diabetes mellitus, smoking history, Zeff, and λHU were not retained in the final multivariable model during the backward stepwise selection process, and hence, Odds Ratios were not applicable to these four variables and were marked with an ellipsis (…).

### 3.3 Predictive Nomogram and External Validation

**Fig. 5.**
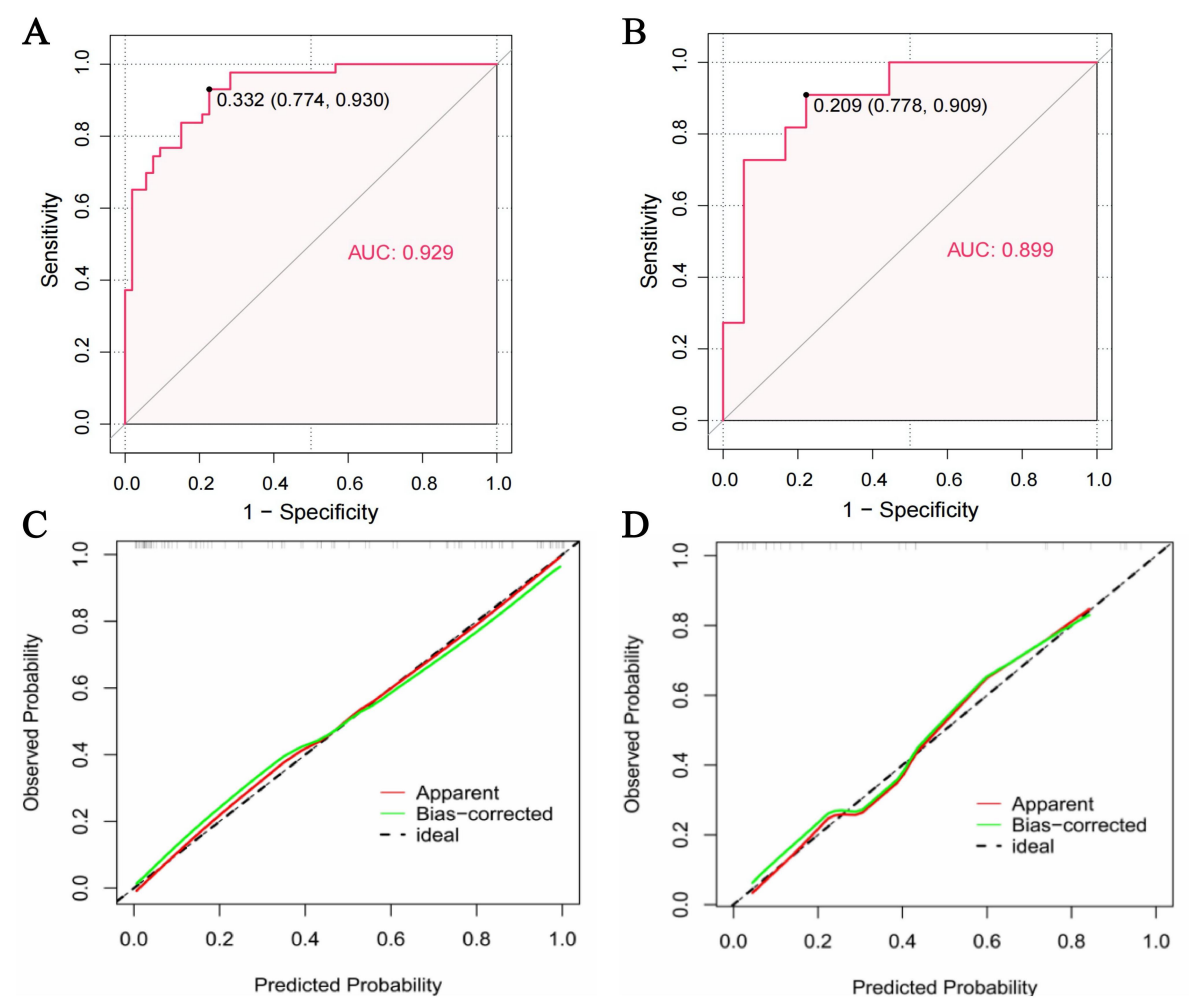
**The performance of the nomogram**. (A,B) Area under curve (AUC) of the model for the identification of cardioembolic (CE) stroke in the study sample (A) and validation cohort (B). The solid black dots indicated optimal cutoff points, with adjacent numerical values representing the corresponding specificity and sensitivity. The model demonstrated strong diagnostic accuracy in identifying CE stroke in both the study sample and the validation cohort. (C,D) Calibration curves of the model in the study sample (C) and validation cohort (D).

**Fig. 6.**
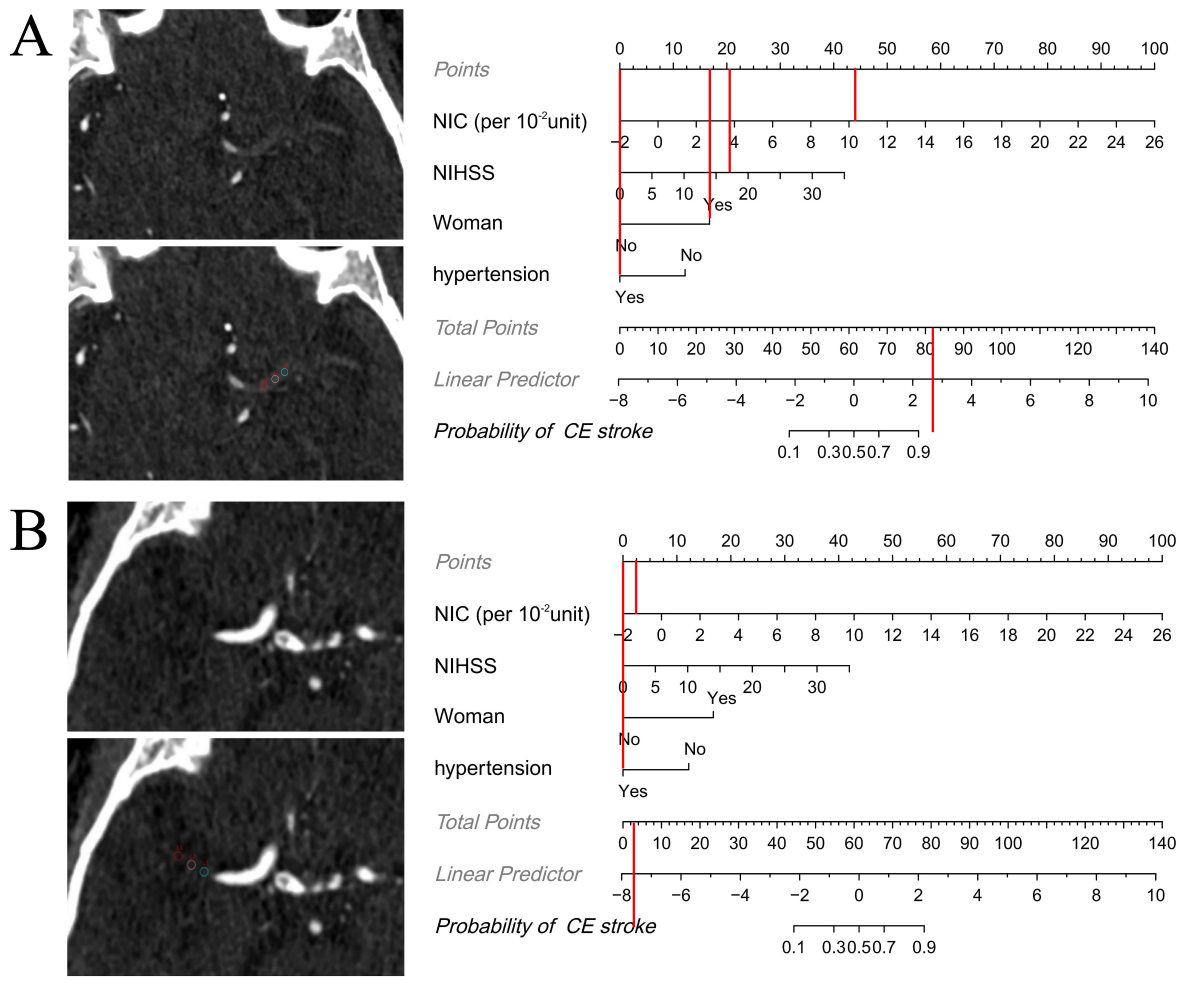
**Examples showing how the nomogram can be applied in clinical practice**. The figures demonstrate the step-by-step process of computing the probability of cardioembolic (CE) stroke using the nomogram. (A) Axial iodine overlay maps in a 77-year-old woman with a National Institutes of Health Stroke Scale (NIHSS) level of 17 at admission. Normalized iodine concentration (NIC) (per 10^-2^ unit) = 10.2, hypertension = “yes”. The total score was 82, which maps to a CE stroke probability of greater than 0.9. The patient had a CE stroke according to Trial of Org 10172 in Acute Stroke Treatment (TOAST), which was consistent with the nomogram result. (B) Axial iodine overlay maps in a 56-year-old man with a NIHSS level of 0 at admission. NIC (per 10^-2^ unit) = –1.4, hypertension = “yes”. The total score was 2.5, which maps to a CE stroke probability of less than 0.1. The patient had an atherosclerotic (LAA) stroke according to TOAST, which was consistent with the nomogram result.

Using the nomogram, the AUC for identifying CE stroke in the study sample was 0.929 (95% CI: 0.881–0.977, *p* < 0.001), while in the validation cohort of 29 patients (Table 1) it was 0.899 (95% CI: 0.784–1, *p* < 0.001). Calibration plots revealed good consistency between the predicted and observed probabilities in the study sample and validation cohort (Fig. 5). Examples of how the nomogram can be applied in clinical practice are shown in Fig. 6.

Using AF, the AUC for identifying CE stroke in the study sample was 0.869 (95% CI: 0.800–0.938), which was lower but not significantly different to the AUC of the nomogram (Z = 1.515; *p* = 0.130).

## 4. Discussion

Determining the source of thrombi in stroke patients is of great value for the selection of secondary prevention and treatment methods [2, 3, 4, 5, 6]. AF is an important diagnostic proof of CE stroke while paroxysmal AF is usually difficult to be detected by emergency ECG at admission because the ECG shows normal results during non-episodic periods of paroxysmal AF. Studies have shown that thrombus permeability parameters assessed on conventional NCCT and CTA scans can help to predict CE stroke [12, 13], but this approach is heavily dependent on accurate image co-registration. In the event that co-registration is unsuccessful, measurement errors are unavoidable [16]. Moreover, the measurement process is complex and time-consuming, thus limiting its clinical utility. On the other hand, DECT can produce iodine overlay maps that directly assess the thrombus permeability, thereby eliminating the image co-registration process [17, 18, 19]. This approach offers greater simplicity, efficiency, and precision in clinical settings. Moreover, DECT can provide additional tissue information due to the acquisition of high- and low-energy data which may be helpful in identifying the source of thrombi [25, 26]. The current study aimed to construct a nomogram model based on DECT to determine the etiology of stroke when the AF history is unknown, or when the stroke is caused by other cardiogenic causes. We found the following: (a) NIC, Zeff, λ_HU_ and baseline NIHSS were all significantly greater in patients with CE stroke than in those with NCE stroke (*p* < 0.001, *p* = 0.009, *p* = 0.007, and *p* < 0.001, respectively). Gender, diabetes mellitus, hypertension and smoking history were each significantly associated with stroke etiology (*p* = 0.002, *p* = 0.018, *p* = 0.022, and *p* = 0.002, respectively); (b) multivariable analysis identified four variables (NIC, gender, hypertension, baseline NIHSS) that were significantly associated with CE stroke (*p* < 0.001, *p* = 0.002, *p* = 0.019, and *p* = 0.003, respectively); (c) The nomogram constructed using these four variables identified CE stroke with an AUC of 0.929 in the study sample and 0.899 in the validation cohort. Calibration plots both revealed good consistency between the predicted and observed probabilities.

Thrombi consist of FP, RBCs, a small number of white blood cells (WBCs), and other components that are cross-linked to form irregular gaps. Together, these constitute the histological basis of thrombus permeability [20]. In agreement with previous studies [12, 13], we observed higher permeability in CE thrombi with greater NIC (CE vs NCE: 0.104 ± 0.043 vs 0.051 ± 0.033, *p* < 0.001). In multivariable analysis, NIC was independently associated with the stroke etiology. Most studies have reported that CE thrombi consist mainly of FP while NCE thrombi consist mainly of RBCs [13, 20, 21, 22]. Fibrin is thought to be attractive to iodide, and the fibrin network is a porous and organized structure that is more conducive to the penetration of contrast agents [27, 28], meaning that thrombi dominated by FP often show high permeability. This may explain why CE thrombi tend to be more pervious. In contrast, less permeability was more frequently observed in thrombi dominated by RBCs, as these cells are firmly bound to each other, making them more impenetrable to contrast agents [29]. Therefore, NCE thrombi are often less pervious. However, a study by Boodt *et al*. [15] suggested that thrombus permeability is not related to stroke etiology. This discord may be explained by the fact that Boodt *et al*. [15] also included patients with small artery occlusion in their study, such as M2 distal segment and M3 segment of the middle cerebral artery, in addition to intracranial proximal artery occlusion. Although the scope of their study was wider than the present work, thrombi in small arteries were too small to place ROIs, hence the measurement error was increased. Furthermore, Boodt *et al*. [15] used traditional permeability parameters that were assessed on NCCT and CTA images, requiring precise co-registration. This is difficult to achieve in clinical practice, leading to uncertainty in the actual measurements. The DECT permeability parameter (NIC) employed in our study was assessed directly on iodine overlay maps without the need for co-registration, and thus more accurately reflected the actual thrombus permeability.

The present study also found that Zeff and λ_HU_ were greater in CE thrombi than in NCE thrombi. The Zeff and λ_HU_ of the thrombus assessed on CTA images represent not only the thrombus itself, but also the contrast agent that penetrated into the thrombus which is a strong indicator of stroke etiology. After adjusting for NIC and clinical variables, no statistically significant association was detected between Zeff (or λ_HU_) and CE stroke. Therefore, we conclude that Zeff and λ_HU_ of the thrombus itself cannot be used to distinguish CE stroke from NCE stroke.

This research also found that CE stroke was more commonly seen in female patients with increased baseline NIHSS and without hypertension. Studies on gender differences in stroke have generally found that women are more likely to suffer from CE stroke, whereas men are more likely to suffer from LAA stroke due to higher rates of smoking and drinking [8, 9]. Zotter *et al*. [10] and Guglielmi *et al*. [11] suggested that CE thrombi were often larger than LAA thrombi, which could easily lead to proximal intracranial occlusions and consequently a higher baseline NIHSS, in agreement with the findings of our study. Hypertension is generally recognized as a risk factor for atherosclerosis, so a high prevalence of LAA stroke, rather than CE stroke, was noted in patients with hypertension. The present study used NIC, gender, baseline NIHSS and hypertension history to develop the nomogram, which was demonstrated to have a good validity and reliability. The AUC of this nomogram was slightly higher than that of AF, although the difference was not statistically significant (0.929 vs 0.869, *p* = 0.130). Therefore, our nomogram can be used to identify CE stroke when the existence of AF is difficult to confirm. Other cardiogenic causes besides AF can also lead to CE stroke, such as patent foramen ovalis. It is easy to be misdiagnosed stroke etiology in this condition when based only on AF. In addition, AF also occurs in some NCE stroke patients. These may explain why the AUC of AF in identifying CE stroke was slightly lower than that of our nomogram.

Timely and rapid identification of CE stroke would greatly assist neuro-interventional physicians in making optimal mechanical thrombectomy plans [30, 31]. Our study used the emergency DECT parameter (NIC) and clinical indicators (gender, baseline NIHSS, and hypertension history) to construct a nomogram that can be a useful tool for identifying CE stroke at admission. At later stages of treatment, some occult AF or other cardiogenic causes are still undetectable, and our nomogram could then also be used to determine the source of thrombi.

There were several limitations to this study. First, only patients with anterior circulation stroke and proximal intracranial occlusions were included. Whether our result is applicable to patients with distal artery occlusion of anterior circulation or posterior circulation requires further study. Second, the sample size of our study was modest due to the limitations of DECT scanning. DECT is an advanced CT equipment capable of performing both conventional and dual-energy scans. Although it is more expensive than traditional CT, DECT offers superior image quality and richer diagnostic information without increasing the radiation dose to patients. With the growing clinical adoption of DECT in recent years, large multicenter DECT studies on stroke will become feasible in the near future. Third, the retrospective study design meant that some clinical data, such as the use of anticoagulants, was missing. This can be overcome by conducting further prospective studies. Fourth, Zeff and λ_HU_ of the thrombus were assessed on DECTA images, which undergo interference from the contrast agent in the thrombus. For this reason our study used NIC, which represents the extent of penetration of the contrast agent, to adjust the results. Further studies using dual-energy NCCT are needed to directly assess the Zeff and λ_HU_ of the thrombus itself. Fifth, we excluded patients with vascular calcifications near the thrombus. This may have introduced selection bias, as it excluded some patients with LAA stroke, thereby potentially skewing the accuracy of the nomogram. However, only patients with intracranial proximal artery occlusion (mainly the M1 and proximal M2 segments of the middle cerebral artery) were included in our study. Vascular calcifications are relatively uncommon in such patients, which may have reduced the potential effect of selection bias on our research findings. Finally, our study lacked histopathological analysis of thrombi, which could have provided important validation of the observed associations between DECT parameters and thrombus composition; further studies are required.

## 5. Conclusions

We demonstrated that a DECT thrombus permeability parameter, NIC, is a useful indicator of CE stroke. A nomogram constructed using NIC, gender, baseline NIHSS and hypertension history showed good diagnostic performance for CE stroke. We believe this nomogram may be especially valuable when the AF history is unknown, or the stroke is caused by other cardiogenic causes.

## Data Availability

The datasets generated or analyzed during the study are mainly human CT images, and are not publicly available due to the ethics policies of the hospital, but are available from the corresponding author on reasonable request.

## Abbreviations

AIS, Acute ischemic stroke; CE, Cardioembolic; NCE, Non-cardioembolic; AF, Atrial fibrillation; ECG, Electrocardiogram; NIHSS, National Institutes of Health Stroke Scale; LAA, Large artery atherosclerosis; NCCT, Noncontrast computed tomography; CTA, CT angiography; DECT, Dual-energy CT; IC, Iodine concentration; NIC, Normalized iodine concentration; RBC, Red blood cell; FP, Fibrin/platelet; Zeff, Effective atomic number; HU, Hounsfield unit; λ_HU_, Slope; SOE, Other determined etiology; DECTA, Dual-energy CTA; GSI, Gemstone Spectral Imaging; WC, Water concentration; ROI, Region of interest; TOAST, Trial of Org 10172 in Acute Stroke Treatment; OR, Odds ratio; AUC, Area under the curve; ROC, Receiver operator characteristic; IQR, Interquartile range; WBC, White blood cell.
